# The Impact of Multiparametric Magnetic Resonance Imaging on Treatment Strategies for Incidental Prostate Cancer after Holmium Laser Enucleation of the Prostate

**DOI:** 10.3390/jcm12144826

**Published:** 2023-07-21

**Authors:** Kwang-Jin Ko, Seongik Choi, Wan Song

**Affiliations:** Department of Urology, Samsung Medical Center, Sungkyunkwan University School of Medicine, Seoul 135-710, Republic of Korea; kwangjin.ko@samsung.com (K.-J.K.); seongik.choi@samsung.com (S.C.)

**Keywords:** benign prostatic hyperplasia, HoLEP, prostate cancer, magnetic resonance imaging, treatment

## Abstract

Purpose: To investigate the impact of multiparametric magnetic resonance imaging (mpMRI) on treatment strategies for incidental prostate cancer (iPCa) after holmium enucleation of the prostate (HoLEP); Methods: Data from 1781 men who underwent HoLEP for clinical bladder outlet obstruction between September 2009 and March 2022 were reviewed retrospectively. Among patients with confirmed iPCa, those with prostate-specific antigen (PSA) levels < 10 ng/mL and who underwent mpMRI 3 months after HoLEP were included. Pathologic results, including Gleason grade (GG) and tumor volume, were identified. mpMRI was interpreted using the Prostate Imaging Reporting and Data System version 2 (PI-RADSv2). Treatment strategies for iPCa according to GG alone, or according to a combination of Gleason grade and mpMRI, were analyzed and compared. Results: Of 1764 men with serum PSA levels < 10 ng/mL, iPCa was confirmed in 64 (3.6%) after HoLEP. Of the 62 men who underwent mpMRI, the median (IQR) age at the time of HoLEP was 72.5 (66.5–78.0) years. The median PSA level and prostate volume were 3.49 (1.82–5.03) ng/mL and 49.6 (38.5–85.4) cm^3^, respectively. The pathologic results of iPCa were as follows: GG1 = 42 (67.7%), GG2 = 13 (21.0%), GG3 = 2 (3.2%), GG4 = 1 (1.6%), and GG5 = 4 (6.5%). Of the patients with GG1 and GG2, 78.6% (33/42) and 53.8% (7/13), respectively, underwent active surveillance (AS). However, of 42 patients with GG1, 27 (64.3%) had a PI-RADSv2 score of 2, and 24 (88.9%) of them underwent AS. Of the 13 patients with GG2, 4 (80%) with a PI-RADSv2 score of 2 underwent AS. All patients with GG 3–5 were clinically expected to have locally advanced PCa and be treated with radiotherapy and/or ADT. Conclusions: For patients with iPCa of GG 1–2 after HoLEP, mpMRI helps to establish a treatment strategy by allowing risk stratification to select those who should be considered for AS or active treatment.

## 1. Introduction

Low urinary tract symptoms due to benign prostatic hyperplasia (BPH) are one of the most common health problems in older men [1]. In the United States, BPH affects about 70% of men in their seventh decade [2]. Holmium laser enucleation of the prostate (HoLEP) is the current standard of care for bladder outlet obstruction (BOO) deemed not appropriate for pharmacological treatment [1,3]. Telemonitoring has recently been applied to the decision making process for planning HoLEP, in addition to questionnaires, to compensate for the differences in uroflowmetry measured at home and in the clinic [4]. Because HoLEP allows for complete removal of adenoma from the transitional zone, it is a useful surgical option for patients with a median lobe or large prostate [5,6]. Even after thorough examination, approximately 5.6–23.3% of incidental prostate cancer (iPCa) cases are identified after HoLEP [7,8]. Most iPCa cases are clinically insignificant or at low risk of progression, but a substantial portion require radical treatment to avoid clinical progression. However, there are no clinical guidelines regarding evaluation of the risk, or appropriate treatment strategies, for men with iPCa.

Recently, multiparametric magnetic resonance imaging (mpMRI) has become a useful tool for the diagnosis and stratification of the risk of prostate cancer (PCa) and clinically significant PCa (csPCa) [9,10,11]. mpMRI interpretation using Prostate Imaging Reporting and Data System version 2 (PI-RADS v2) with a 5-point scale is significantly associated with histologic features such as cribriform variant status and perineural invasion after robot-assisted radical prostatectomy (RARP) [12]. Previous studies have report that mpMRI can be used for active surveillance of iPCa [13] because mpMRI helps discriminate between indolent and high-grade tumors, thus identifying clinical progression [14]. In addition, mpMRI should be considered for men with elevated prostate-specific antigen (PSA) levels to detect csPCa scheduled for HoLEP [3]. The overall sensitivity, specificity, negative predictive value, and positive predictive value of mpMRI for csPCa were reported to be 0.94, 0.63, 0.92, and 0.67, respectively [15]. However, most mpMRI studies have excluded patients with a history of prostate surgery. Therefore, no studies have examined the role of mpMRI in the management of iPCa confirmed after HoLEP.

Here, we investigate the role of mpMRI in developing treatment strategies for iPCa after HoLEP. Treatment according to Gleason grade, or according to a combination of Gleason grade and mpMRI, was analyzed and compared.

## 2. Materials and Methods

### 2.1. Study Design

This study was approved by the Institutional Review Board (IRB) of our institution (Samsung Medical Center IRB No. 2023-06-040). The IRB waived the informed consent due to the retrospective study design. All study protocols were in accordance with the principles of Declaration of Helsinki.

**Figure 1:**
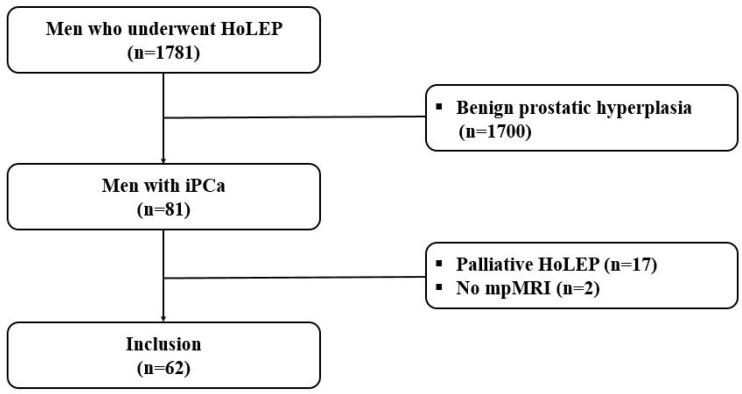
Flow chart showing patient selection.

The records of 1781 men who underwent HoLEP for clinical BOO between September 2009 and March 2022 were reviewed retrospectively. The inclusion criteria were as follows: (1) pathologically confirmed iPCa after surgery, (2) serum PSA levels < 10 ng/mL, (3) and mpMRI performed 3 months after surgery. The exclusion criteria were as follows: (1) men who had undergone prostate surgery or biopsy, (2) were treated with a 5α-reductase inhibitor, and (3) had acute prostatitis or urinary tract infection. Men with a pathological diagnosis of BPH (n = 1700), men who underwent palliative HoLEP (n = 17), and men who did not undergo mpMRI (n = 2) were excluded. Finally, 62 men with iPCa after HoLEP were included in the final analysis (Figure 1).

### 2.2. Data Collection

The medical records of all men were reviewed at the time of HoLEP and data (age at surgery, PSA level, body mass index (BMI), and prostate volume) were extracted. Transrectal ultrasound (TRUS) was performed and prostate volume was calculated by applying the ellipsoid formula: width × length × height × 0.52. PSA density (PSAD) was calculated as total PSA/prostate volume.

At 3 months post HoLEP, mpMRI was performed using a 3.0-T MRI instrument (Intera Achieva TX, Philips Healthcare, Best, The Netherlands) with a 6-channel, phase-array body coil. The scanning protocol for mpMRI included T1-weighted, T2-weighted, and diffusion-weighted imaging, with b values of 0, 100, 1000 and 1500 s/mm^2^, and dynamic contrast-enhanced imaging after intravenous injection of a gadolinium diethylenetriamine penta-acetic acid (Gadovist, Schering, Berlin, Germany), according to the European Society of Uro-genital Radiology (ESUR) guidelines [16]. All images were loaded using a picture archiving and communication system (Centricity, GE Healthcare, Barrington, IL, USA), and interpreted by two uro-radiologists with more than 15 years of experience of prostate MRI. The reviewers scored an index lesion according to the PI-RADS v2 using a 5-point scale [17].

All surgical specimens were examined and interpreted by a uro-pathologist with more than 15 years of experience. iPCa was reported according to the guidelines of the International Society of Urological Pathology (ISUP) consensus conference (2014) [18]. Gleason grade (GG) 1 is equivalent to a Gleason score (GS) of ≤3 + 3 = 6, GG2 is equivalent to GS 3 + 4 = 7, GG3 is equivalent to GS 4 + 3 = 7, GG4 is equivalent to GS 4 + 4 = 8, and GG5 is equivalent to GS 9–10 [19]. Tumor involvement was estimated by assessing the percentage tumor involvement within the entire surgical specimen.

### 2.3. Statistical Analysis

The clinical characteristics of patients were assessed using descriptive statistics. Quantitative variables were reported as the median (range) or mean (standard deviation), and qualitative variables as absolute values (percentages). All statistical analyses were performed using IBM SPSS statistics for Windows, version 23.0 (IBM Corp., Armonk, NY, USA). A *p* value of <0.05 was considered significant.

## 3. Results

### 3.1. Baseline Demographic and Clinicopathologic Characteristics

Of 1764 men with serum PSA levels < 10 ng/mL, iPCa was confirmed in 64 (3.6%) after HoLEP. Table 1 summarizes the baseline characteristics of 62 of the men with confirmed iPCa who underwent mpMRI. The median (IQR) age was 72.5 (66.5–78.0) years. The median total PSA level and prostate volume were 3.49 (1.82–5.03) ng/mL and 49.6 (38.5–85.4) cm^3^, respectively. PI-RADSv2 score 3, 4, and 5 lesions were identified in 14 (22.6%), 11 (17.7%) and five (8.1%) patients, respectively. T1a (defined as cancer in less than 5% of the removed tissue) was identified in 40 (64.5%) patients, and 22 (35.5%) had T1b (cancer in ≥5% or the removed tissue). The mean (SD) total International Prostate Symptom Score (IPSS) was 20.6 (5.2).

Table 2 summarizes the pathologic results of iPCa after HoLEP: GG1 = 42 (67.7%), GG2 = 13 (21.0%), GG3 = 2 (3.2%), GG4 = 1 (1.6%), and GG5 = 4 (6.5%). The median percent tumor volume was 2.0 (1.0–5.0)%.

### 3.2. Treatment Strategy for iPCa

**Figure 2:**
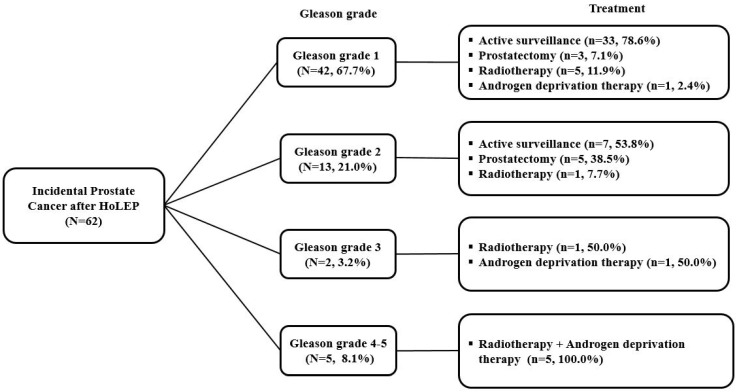
Treatment strategy for patients with iPCa after HoLEP according to Gleason grade. iPCa, incidental prostate cancer; HoLEP, holmium laser enucleation of the prostate.

Figure 2 depicts the treatment strategy for iPCa after HoLEP according to the GG. AS was performed for 78.6% (33/42) and 53.8% (7/13) of patients with GG1 and GG2, respectively. All patients with GG 3–5 received definite treatment (radiotherapy and/or ADT).

The treatment strategy for patients with iPCa after HoLEP, based on the combination of GG and mpMRI results, is shown in Table 3. Of 42 patients with GG1, 27 (64.3%) had a PI-RADSv2 score of 2, and 24 (88.9%) of these underwent AS. In addition, 66.7% of patients with a PI-RADSv2 score of 3 or 4 (6/9 and 4/6, respectively) underwent AS. Of the 13 patients with GG2, when the PI-RADSv2 score was 2, AS was performed in 4 (80%) patients. In addition, when the PI-RADSv2 score was 3 or 4, 40.0% (2/5) and 33.3% (1/3) of patients underwent AS, respectively. All patients with GG 3–5 were clinically expected to have locally advanced PCa that required radiotherapy and/or ADT.

## 4. Discussion

In this study, we analyzed the role of mpMRI in the treatment of iPCa after HoLEP. Among a cohort of 1764 men, iPCa was confirmed in 64 (3.6%) after HoLEP. Based on GG alone, 78.6% (33/42) of GG1 patients and 53.8% (7/13) of GG2 patients underwent AS. However, when the treatment strategy was planned according to the combination of GG and mpMRI, 88.9% (24/27) of patients with GG1 and a PI-RADSv2 score of 2, and 80.0% (4/5) with GG2 and a PI-RADSv2 score of 2, underwent AS. Therefore, mpMRI can be used for risk stratification of iPCa, and can help patients and clinicians decide on the appropriate treatment strategy. To the best of our knowledge, this study is the first study to evaluate the role of mpMRI for risk stratification of iPCa after HoLEP.

Previous studies report that most iPCa cases after HoLEP are low-risk or indolent disease [20,21]; thus, these cases are reliable candidates for AS. However, we found that 32.3% (20/62) of patients with iPCa had csPCa, defined as GG ≥ 2. In addition, patients with GG3–5 (11.3%, 7/62) had a PI-RADSv2 score 4–5 on mpMRI, suggestive of locally advanced PCa. However, in the real-world setting, there is no clinical consensus regarding the most appropriate treatment of these patients. Therefore, it is necessary to stratify the risk of iPCa, and to determine the treatment strategy accordingly.

PSA is the most widely used serum biomarker for PCa screening. Bohjani N et al. reported that iPCa was diagnosed in 8.1% (103/1272) of patients after HoLEP, and that preoperative PSA levels were significantly associated with iPCa (OR: 1.03; 95% CI, 1.01–1.05; *p* < 0.001) [2]. In addition, Elkoushy M. A. et al. examined 1242 patients who underwent HoLEP, and found that total PSAD was an independent predictor of iPCa (OR: 3.62; 95% CI, 1.81–5.12; *p* = 0.03) [22]. However, when the PSA level is <10 ng/mL, the PSA-guided approach does not reliably predict iPCa (PSA is not a very specific marker for PCa) [23]. In our study, we found no difference in the PSA levels between the BPH and iPCa groups after HoLEP (3.41 vs. 3.49, respectively; *p* = 0.816). In addition, Magistro G et al. analyzed 1125 men treated with HoLEP, and found that PSA and PSAD were not significantly associated with iPCa in patients with prostates larger than 100 cc (all *p* > 0.05) [8].

Detection of csPCa is a major challenge because it affects the clinical decision to undertake AS or active treatment. In our entire cohort, 64.5% (40/62) of patients underwent AS, while 35.5% (22/62) received active treatment. Specifically, AS was undertaken for 78.6% (33/42) of patients with GG1 and for 53.8% (7/13) with GG2. However, previous studies report that mpMRI can detect csPCa [24], or improve the selection of AS candidates significantly [25]. Therefore, when we categorized patients according to the combination of GG and mpMRI results, 27 (64.3%) of 42 patients with GG1 had a PI-RADSv2 score of 2; of these, 24 (88.9%) underwent AS. In addition, four (80%) of five patients with GG 2 and a PI-RADSv2 score 2 underwent AS. These results indicate that the combination of GG and mpMRI results plays an important role in risk stratification of iPC, and helps the decision regarding treatment strategy. Our results are supported by a study showing that iPCa patients with invisible mpMRI results should be considered for AS, because the risk of harboring residual cancer is low [26]. Conversely, all patients with GG 3–5 had a PI-RADSv2 score of 4–5, suggestive of locally advanced PCa; these patients received definitive treatment.

Of the 40 patients who were planned for AS, 39 (97.5%) maintained AS during a median follow-up of 42.7 months, whereas 1 (2.5%) underwent radical prostatectomy (RP) at 47 months due to concerns about progression. Of the total eight patients who underwent RP, one had no residual tumor, and an upgraded GG was confirmed in another (12.5%) patient. None of the patients experienced biochemical recurrence during a median follow-up of 78.5 months. Of the remaining 14 patients who were treated with radiotherapy and/or ADT during a median follow-up of 40.2 months, 1 patient who received radiotherapy underwent additional ADT due to PSA elevation at 22 months. None of the patients died of prostate cancer during a median follow-up of 72.8 months. Collectively, a very favorable oncologic outcome can be expected if iPCa is risk-stratified and treated appropriately.

Klein C et al. examined the predictive factors for iPCa progression after HoLEP, and reported that post-operative PSA level was significantly associated with iPCa progression (OR: 2.35, *p* < 0.001) [27]. In particular, post-operative PSA ≥ 2 ng/mL was the only predictor of iPCa progression, thus requiring close monitoring or early definite treatment. Therefore, continuous PSA monitoring after HoLEP is important for early prediction of iPCa progression. In our study, of the 40 patients who were planned for AS, the median PSA (IQR) level before HoLEP was 3.59 (1.83–5.05) ng/mL, and the median post-operative PSA was 0.62 (0.31–1.04) ng/mL. One patient’s PSA level was 2.06 ng/mL (pre-operative PSA: 7.21 ng/mL) after HoLEP, but there was no evidence of radiologic progression during the follow-up of 38.0 months.

The results of our study show that mpMRI can be used for the risk stratification of iPCa after HoLEP; however, there are some caveats with respect to interpretation of mpMRI findings after HoLEP. First, changes in the normal anatomical structure after HoLEP, such as gland deformity or residual adenoma, can make interpretation of mpMRI difficult, confounding the distinction from csPCa [28]. Second, hemorrhage inside the prostate can reduce the T2 signal intensity, making it difficult to distinguish it from PCa. Therefore, an interval of more than 6 weeks is generally recommended to allow hemorrhage absorption [29]. Third, prostatitis is reported in approximately 7% of cases after HoLEP, and this should be considered when interpreting mpMRI, because the radiologic features are similar to those of csPCa. In our study, we tried to reduce the ambiguity of interpretation by leaving an interval of 3 months from HoLEP to mpMRI. As a whole, when interpreting mpMRI after prostate surgery, it should be considered that the risk of csPCa may be about 5–10% lower than that of naïve prostate [28].

Despite its clinical implications, this study has several limitations. First, it was a retrospective study of data from a single institution, meaning that there is a possibility of inherent selection bias; however, we analyzed prospective data held in databases to evaluate real-world clinical practice. Second, although the number of patients with iPCa was relatively small, to the best of our knowledge, this study examined the largest number of HoLEP cases collected to date. Third, none of the patients included in our study underwent an additional prostate biopsy based on the mpMRI results. Instead, treatment strategies were planned according to the mpMRI results; therefore, upgrading of the GG and/or the presence of csPCa could not be confirmed. Therefore, these data should be confirmed in a large prospective study.

## 5. Conclusions

Here, we confirmed iPCa in 3.6% of patients after HoLEP, among whom iPCa of GG 2 or higher was identified in 32.3%. We comprehensively analyzed the treatment strategy for iPCa after HoLEP according to the combination of the GG and mpMRI results. In patients with iPCa of GG1–2, mpMRI helps to establish a treatment strategy through risk stratification to select those who should be considered for AS or active treatment. A favorable oncologic outcome can be expected after individualized treatment. Further studies are needed regarding interpretation of mpMRI after HoLEP, and to establish a systemic plan for the management of iPCa in combination with other clinical tools.

## Figures and Tables

**Table 1:** Baseline characteristics.

Variable	*p*
No. of patients, n (%)	62 (100)
Age, years	
Median (IQR)	72.5 (66.5–78.0)
Body mass index, kg/m^2^	
Median (IQR)	24.6 (22.8–26.3)
Total PSA, ng/mL	
Median (IQR)	3.49 (1.82–5.03)
Prostate volume, cm^3^	
Median (IQR)	49.6 (38.5–85.4)
PSA density, ng/mL/cm^3^	
Median (IQR)	0.06 (0.03–0.09)
PI-RADS v2 score, n (%)	
1–2	32 (51.6)
3	14 (22.6)
4	11 (17.7)
5	5 (8.1)
T stage, n (%)	
T1a	40 (64.5)
T1b	22 (35.5)
IPSS total	
Mean (SD)	20.6 (5.2)

IQR, interquartile range; PSA, prostate-specific antigen; PI-RADSv2, Prostate Imaging Reporting and Data System version 2; IPSS, International Prostate Symptom Score; SD, standard deviation.

**Table 2:** Pathologic results of incidental prostate cancer after holmium laser enucleation of the prostate.

Variable	Value
Gleason grade, n (%)	62 (100)
GG1	42 (67.7)
GG2	13 (21.0)
GG3	2 (3.2)
GG4	1 (1.6)
GG5	4 (6.5)
Tumor volume, %	
Median (IQR)	2.0 (1.0–5.0)

GG, Gleason grade; IQR, interquartile range.

**Table 3:** Treatment strategy for patients with iPCa after HoLEP according to the combination of GG and mpMRI results. iPCa, incidental prostate cancer; HoLEP, holmium laser enucleation of the prostate; PI-RADS, Prostate Imaging Reporting and Data System.

Incidental Prostate Cancer after HoLEP			
Gleason Grade (n, %)	mpMRI Results (n, %)	Clinical T Stage (n, %)	Treatment (n, %)
Gleason grade 1 (42, 67.7%)	PI-RADS 2 (27, 64.3%)	cT1 (27, 100.0%)	Active surveillance (24, 88.9%)
			Radical prostatectomy (2, 7.4%)
			Radiotherapy (1, 3.7%)
	PI-RADS 3 (9, 21.4%)	cT1 (2, 22.2%)	Active surveillance (1, 50.0%)
			Radical prostatectomy (1, 50.0%)
		cT2a (6, 66.7%)	Active surveillance (5, 83.3%)
			Radiotherapy (1, 16.7%)
		cT2c (1, 11.1%)	Radical prostatectomy (1, 100.0%)
	PI-RADS 4 (6, 14.3%)	cT2a (2, 33.3%)	Active surveillance (2, 100.0%)
		cT2b (1, 16.7%)	Active surveillance (1, 100.0%)
		cT2c (2, 33.3%)	Active surveillance (1, 50.0%)
			Radical prostatectomy (1, 50.0%)
		cT3a (1, 16.7%)	Hormone therapy (1, 100.0%)
Gleason grade 2 (13, 21.0%)	PI-RADS 2 (5, 38.5%)	cT1 (4, 80.0%)	Active surveillance (4, 100.0%)
		cT2a (1, 20.0%)	Radical prostatectomy (1, 100.0%)
	PI-RADS 3 (5, 38.5%)	cT2a (4, 80.0%)	Active surveillance (2, 50.0%)
			Radical prostatectomy (2, 50.0%)
		cT3a (1, 20.0%)	Radiotherapy (1, 100.0%)
	PI-RADS 4 (3, 23.0%)	cT2a (1, 33.3%)	Radical prostatectomy (1, 100.0%)
		cT2c (2, 66.7%)	Radical prostatectomy (1, 50.0%)
			Active surveillance (1, 50.0%)
Gleason grade 3 (2, 3.2%)	PI-RADS 4 (1, 50.0%)	cT3a (1, 100.0%)	Radiotherapy (1, 100.0%)
	PI-RADS 5 (1, 50.0%)	cT3a (1, 100.0%)	Hormone therapy (1, 100.0%)
Gleason grade 4–5 (5, 8.1%)	PI-RADS 4 (1, 20.0%)	cT3b (1, 100.0%)	Radiotherapy + hormone therapy (1, 100.0%)
	PI-RADS 5 (4, 80.0%)	cT3b (4, 100.0%)	Radiotherapy + hormone therapy (4, 100.0%)

## Data Availability

The dataset used and/or analyzed during the current study is available from the corresponding author upon reasonable request. The data are not publicly available due to privacy concerns.
